# Nanoparticle technology and its implications in endodontics: a review

**DOI:** 10.1186/s40824-020-00198-z

**Published:** 2020-12-04

**Authors:** Natasha Raura, Anirudh Garg, Arpit Arora, M. Roma

**Affiliations:** Manipal College of Dental Sciences, Mangalore, Manipal Academy of Higher Education, Manipal, Karnataka India

**Keywords:** Antibacterial, Dentistry, Nanoparticles, Nanodentistry, Endodontics

## Abstract

**Background:**

The era of Nanomaterials has had a long lasting impression in the field of medical science. It’s excellent use in medicine has led to its application in dental science. Serious concerns regarding the eradication of microbial biofilms from the root canal system still exists in the field of endodontics. Nanoparticles have proven to be much more efficient with good bonding capabilities and surface chemistry as compared to the conventional materials. The practical applications of nanotechnology in endodontics has led to future prospects in research in this field.

**Main body:**

Nanoparticles in endodontics have shown promising results. The various nanoaprticles like graphene, silver nanoparticles, chitosan, hydroxyapaptite nanoparticles, Iron compound, zirconia, Poly (lactic) co-glycolic acid, bioactive glass, mesoporous calcium silicate, titanium dioxide nanoparticles, Magnesium, Calcium oxide and Copper oxide have been discussed. These nanoparticles have fetched and shown great results in various application in endodontics like incorporation of nanoparticles in selaers, obturating materials, irrigation, and intracanal medicament.

**Conclusion:**

The application of nanoparticles from natural and synthetic materials is rapidly evolving in dentistry. These biomaterials have helped in treatment of oral diseases, in eradication of smear layer and biofilms, have been incorporated in various dental materials for their antimicrobial effects. Combining all their beneficial aspects, these nanoparticles will provide new paradigm shift in dentistry. This review on nanoparticles will provide the reader with the latest knowledge of these materials, their mechanism of action and its implications in endodontics.

## Introduction

Majority of microorganisms are inhabitants of the oral cavity [1]. Endodontic disease is a biofilm facilitated infection, and the fundamental objective in its treatment is the removal of these biofilms from the endodontic canals. Biofilms have been defined as ‘aggregates of microorganisms in which cells are frequently embedded in a self-produced matrix of extracellular polymeric substances (EPS) that are adherent to each other and/or a surface [2]. Various studies have been performed that focused on antibacterial means to remove this problem, but most of the studies were unsuccessful in achieving desired results because of the rapid release and degradation of antibacterial agents leading to inefficiency and safety alarms [3, 4].

To overcome the drawbacks of the conventional antibacterial agents and to achieve promising results in endodontics, antimicrobial nanoparticles offering numerous advantages like large surface-area-to-volume ratio, ultra-small sizes, and excellent chemical and physical properties have been introduced [3].

As per the European Commission’s Recommendation “nanomaterial” is defined as a natural, incidental, or manufactured material containing particles, in an unbound state or as an aggregate or as an agglomerate and where, for 50% or more of the particles in the number size distribution, one or more external dimensions is in the range 1–100 nm [5].

Nanoparticles (NPs) provide a new advancement for the prevention and treatment of dental infections. The positive charge and increased surface area of NPs allow them to react with the negatively-charged bacterial cells causing increased antibacterial activity [6]. Furthermore, NPs can be combined polymers or can be coated onto biomaterial surfaces. This was also found to exhibit enhanced antimicrobial property [3].

## History

Nanoparticles have had a long history linked with modern science [7]. The concept of nanotechnology was first explained by Dr. Richard Feynman in 1959. In 1991, Dr. Sumio Lijima introduced the concept of nanotubes. The term ‘nano-dentistry’ was coined by Dr. Freitas Jr. in the year 2000. He developed nanomaterials and nanorobots, helped in regeneration of dentition, and developed dentifrobots – robots in dentrifices. All these ideas were initially considered impossible and were termed as “science fiction”, but in the current era, they are being finally recognized by the clinicians [8].

## Classification (Fig. 1)

The classification of nanoparticles is as follows:

**Fig. 1:**
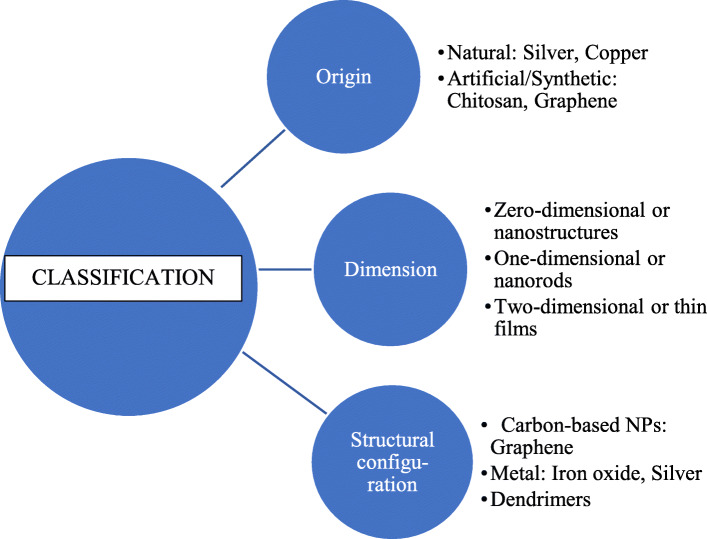
Classification of Nanoparticles

## Benefit of nanoparticles

The NPs as antibacterial agents utilizes various mechanisms which are different to antimicrobial mechanisms of other conventional therapies. As nanoparticles are less stable, exhibit weaker bonding and interaction with other molecules, they provide greater benefits. Furthermore, due to the high surface area to volume ratio the energy statistics of the particles is established [9]. NPs are also involved in disruption of cell wall synthesis, inhibition of various enzymes, for instance DNA gyrase and DNA-dependent RNA polymerase. Moreover, to reap better rewards from NPs, their mechanical, optical, chemical and electrical properties can be modified.

## Mechanism of action

A few known mechanisms by which NPs act are described below:

### Electrostatic interaction leading to cell membrane disruption

As negative and positive charge attract each other, the positively charged nanoparticles react with the negatively charged surface of microorganisms leading to their accumulation of NPs on the bacterial cell surface. These positively charged NPs are bonded effectively to the cell membrane leading to upsetting of the cell wall framework which leads to an increase in the permeability of the cell allowing the entry of more and more NPs into the bacteria, causing cellular content leakage. These NPs by binding to mesosomes, affect respiration, division, also DNA replication [10, 11].

### Metal ion homeostasis

Metabolic functions are measured which are largely dependent on hemostasis of metal ion present in the microbes. An irreversible damage causing retardation of growth or killing of the microbe is caused by excess of metal NPs, which disrupt this important function [12]..

### Production of reactive oxygen species

NPs gain access to the cell membrane of the microorganism and cause the release of ROS, an oxidative stress occurs in the cell which initiates an attack on the microbe. Due to this attack, respiration and production of ATP is decreased, this causes disruption of the cell membrane. The formation of ROS by a metal oxide occurs by active redox cycling and by the pro-oxidant functional group on metal oxide-NP interface [13]..

### Protein and enzyme dysfunction

NPs cause a formation of carbonyls which are protein bound in nature by catalysing the oxidative process of amino acid chain resulting in degradation of protein, inactivation of various enzymes and disruption of catalytic activity [14, 15].

### Genotoxicity and inhibition of signal transduction

Due to their electrical properties, NPs interact with the nucleic acid molecules leading to a negative influence on the process of chromosomal and plasmid DNA replication which results in signal transduction inhibition [16–18].

## Nanoparticles in dental application

Due to the above mentioned properties, benefits over other conventional materials and mechanism of action, there has been an enormous increase in the application of nanoparticles in various fields of dentistry since their introduction. These nanoparticles can be incorporated in a sealer, obturating material, intracanal medicament and irrigating solutions to provide the desired results.

### Irrigation

Root canal irrigation is described as “washing by a stream of fluid” whereby “intracanal irrigation facilitates physical removal of materials from the canal and introduction of chemicals for antimicrobial activity, demineralization, tissue dissolution, bleaching, deodorizing and hemorrhage control” [19]. The most commonly used irrigants are chlorhexidine (CHX), ethylenediaminetetraacetic acid (EDTA) and sodium hypochlorite (NaOCl).

Sodium Hypochlorite is a regularly used material that accomplishes most of the ideal properties of an irrigant. It dissolves organic and inorganic tissue, also causes oxidation and hydrolysis of cellular proteins. However, it’s cytotoxicity and impediments due to the accidental extrusion beyond the apical foramen of the tooth is of major concern [20].

CHX is reasonably safer than NaOCl but the ability for the removal of the biofilm or smear layer in root canal dentine is not adequate.

Knowing the limitations of conventional irrigants, nanoparticles have been introduced at developing novel irrigation materials. Chitosan nanoparticles have showed enhanced antibiofilm efficacy, and has the potential to disable bacterial endotoxins. These nanoparticles cause enhanced bacterial degradation demonstrated by an organized release of singlet oxygen species. They are suggested for usage as a finishing rinse in irrigation of root canals as they are non-toxic to eukaryotic cells [21].

### Interappointment Intracanal medicaments

Intracanal medicaments function as anti- inflammatory and antibacterial agents which can be used in between appointments. They are available as pastes, gels, or points that are introduced into the canal.

Calcium hydroxide paste is the most commonly used material. It initiates the release of hydroxyl ions that increases the pH within the root canal distressing the DNA, cytoplasmic membranes, and enzymes of microorganisms. Silver nanoparticle (size 20 nm) can be mixed with calcium hydroxide, which showed the increased antibacterial action when calcium hydroxide is used alone or in combination with chlorhexidine [22]. A commercially available product NanocarePlus Silver and Gold (NanoCare Dental, Nanotechnology, Katowice, Poland) has shown promising antimicrobial properties as an intracanal medicament.

### Obturation

Obturation is the procedure of filling a canal three dimensionally, after it has been chemo-mechanically prepared and disinfected. A bulk filler (solid or semisolid) is used alongside a sealer in an attempt to do so.

#### Bulk filler

The commonly used bulk fillers for the process of obturation are Gutta percha (GP), silver points, and Resilon. GP is an obturating material which is biocompatible, inert and structurally stable. Latest preparations have assimilated nanoparticles and bioglass, to obtain oroactive properties from GP. It was reported by Lee et al. that nano-diamond GP (NDGP) composite embedded with amoxicillin resulted in superior mechanical properties (like strength and elastic modulus) over the routinely used GP [23].

### Sealers

The combination of Endodontic sealers used along with obturating materials is an essential step to achieve a good three dimensional seal in the root canal system. Even though, GP is heated in the root canal to increase its flowability, it still cannot be held to the root dentin. Thus, due to this drawback of GP obturating material, a sealer is required to fill the gaps occuring between the obturating material and root dentine in order to achieve a fluid- snug seal. A study was performed by Kishen et al. in which chitosan and zinc oxide nanoparticles were incorporated in obturating sealers. The results showed that these NPs inhibited bacterial penetration in the canal which led to a conclusion that inclusion of these NPs in the sealers resulted in a successful outcome [24]. Zinc Oxide has been synthesized as a nanoparticle for applications as a sealer and is commercially available as NanoSeal-S (Prevest DenPro). Del Carpio-Perochena et al. later in his study concluded that chitosan loaded endodontic sealers maintained their anti-bacterial efficacy for a longer time [25].

## Various nanoparticles used in Endodontics

### Organic nanoparticles

#### Graphene

Graphene, an allotrope of carbon is the thinnest material which forms an even crystal lattice exclusive of any structural dislocations. This NP is used for diagnosis and detection of disease and formation of anti-bacterial surfaces [26]. Sodium hypochlorite has been used as an intracanal irrigant because of its potent antimicrobial and tissue-dissolving capabilities. But one of its main disadvantage as an irrigant is it causes rapid hemolysis and soft tissue ulceration if extruded apically. By incorporating graphene into silver nanoparticles, the antibacterial property remained the same however, cytotoxic effects to bone and soft tissues showed reduction [27].

He et al. in a study investigated the antimicrobial efficacy of Graphene oxide NPs against common pathogens like S. mutans and concluded that these nanoparticles were extremely effective in killing the growth of S. mutans.

Graphene nanoplatelet, a derivative of Graphene has also shown antimicrobial properties against various microorganisms especially S. mutans in a study performed by Rago et al. The SEM images have shown that, a strong mechanical bond exists between the graphene nanoplatelet and cells which involves shrinking and trapping of cells ultimately leading to the death of these microorganisms [28].

#### Chitosan

Chitosan is a deacetylated derivative of chitin, and is the second most abundant natural biopolymer and can be modified chemically. Chitosan has shown brilliant antimicrobial, antifungal and antiviral characteristics. The mechanism of action of Chitosan NPs is based on the principle of electrostatic interaction leading to cell membrane disruption. This results in increased permeability of cell wall, eventually causing cell death and microleakage of its intracellular components [29].

Kishen et al. were the first in the field of Nanoparticles to evaluate the root canal disinfection by using Chitosan NPs. Chitosan can be penetrated in the complexities of the root canal and dentinal tubules, thus eliminating microorganisms based on its concentration and time-dependent property even after 3 months [23].

Barreras US et al. in his in-vitro study used Chitosan Nanoparticles along with CHX in order to remove *Enterococcus faecalis* from the canals. This combination also resulted in the formation of membrane barriers at the peri-radicular area [30].

#### Poly (lactic) co-glycolic acid

Poly **(**lactic) co-glycolic acid Nanoparticles incorporated with photoactive drugs are used as an essential adjunct in the eradication of microorganisms from endodontic canals. The combination of these methylene blue filled NPs and light are used to reduce microbial counts adhered to the root dentin and canals. It is one of the most important NPs used in the application of endodontics [31].

### Non-organic nanoparticles

#### Bioactive glass nanoparticles

SiO2, Na2O, and P2O5 at altered concentrations form the main components of Bioactive glass based NPs. Their size ranges from 20 to 60 nm in size.

It’s advantages include:

- Alkaline pH: pH is increased due to the release of ions in an aqueous environment
- Osmotic effects: Presence of high osmotic pressure more than 1% is fatal for many microorganisms.
- Calcium-Phosphate precipitation: Results in mineralization of the demineralized enamel surface.
- Highly amorphous in nature

Waltimo et al. after performing their in vitro study using the ideal preparation of Bioactive Glass solutions for the disinfection of root canals stated that the combination of high pH orientation with a constant flow of alkaline materials proved to be more effective [32].

#### Mesoporous calcium silicate

These are NPs with size ranging from 80 to 100 nm having high specific surface area and pore volume ratio. These NPs find its use in filling of apical third of the root canals due to its property of being highly viscous in nature [33]. Its other advantages in Endodontics include:

- Drug delivery
- Antibacterial efficiencies
- Injectability
- Apatite mineralization
- Osteo-stimulation

#### Hydroxyapatite nanoparticles

Although Hydroxyapatite NPs have been used commonly in the field of medicine and dentistry, its use in endodontics has been questionable. The main function of HAp particles is to integrate into the dentinal tubules and to seal their opening which helps in preventing the exposure of nerves to obnoxious external stimuli. Hence, HAp finds its application in decreasing dentin hypersensitivity. These nanocrystals are present in various dentrifices and mouthrinsing solutions which helps to remineralize the demineralized enamel surface [34]. HAp is a highly biocompatible material capable of binding to bone thus reducing any local or systemic inflammatory reaction. Thus, it can be used as a periapical healing agent [34, 35].

### Metal nanoparticles

#### Silver nanoparticles (AgNP’s)

AgNP’s finds its applications in various fields of dentistry with endodontics being an emerging field. They can easily penetrate the bacterial cell membrane due to their larger surface area and small size causing rapid bactericidal action. It is biocompatible, shows low bacterial resistance, low toxicity [36], and longstanding antibacterial activity [37]. Biologically produced silver NPs have shown an effective antibacterial property against *Enterococcus faecalis*. Discoloration is one of the drawbacks of using AgNPs in endodontic treatment of anterior teeth.

In a study performed by Afkhami et al. comparing the irrigation properties of 100 ppm silver nanoparticles with 2.5% sodium hypochlorite, it was concluded that silver nanoparticles resulted in superior antibacterial effect. It was also reported that Poly vinyl coated Silver nanomaterials resulted in reduced cytotoxicity when compared to Sodium hypochlorite.

#### Silica nanoparticles

Silica Nanoparticles have had a positive impact in the field of dentistry, more so in conservative dentistry than in Endodontics. These nanoparticles have shown excellent biocompatibility and large surface area with low levels of toxicity and density. They are widely used as dental fillers in various restorative materials, and also as a polishing agent due to it’s ability to produce lower roughness of the polished substrate [38].

### Metal oxide nanoparticles

#### Iron compound (FeOx)

Iron compound (FeOx) nanoparticle finds its importance in biological and medicinal field [34]. The use of antibiotics in the elimination of endodontic biofilms is difficult due to the production of exopolymers, which is not permeable to antibiotics and certain immunological cells. Thus iron based NPs plays a role in complete elimination of these microorganisms. Iron- oxide nanoparticles are also applicable in the eradication of biofilms present on dental implants [39].

#### Zirconia

Zirconia, a chemical oxide, has been considerably used in dentistry due to its optical and metallic properties similar to the tooth. ZrO_2_ is proven to be a high-performance ceramic material due to its high toughness, strength, corrosion resistance and great chemical properties. It has shown to eradicate bacterial colonization with low cytotoxic effects due to its property of insolubility in water [40, 41]. Zirconia based NPs are highly potent against specific microorganisms such as *E. faecalis* and thus, widely used as an anti-microbial agent in endodontics [31].

Mineral trioxide aggregate (MTA) is the most commonly used agent for root end filing and direct or indirect pulp capping. Portland cement is the main constituent of MTA. Tanamaru et al. in their study found that Zirconia NPs can be used as an effective radiopacifier as a supplement in Portland cement without negatively impacting its biocompatibility. Two groups of micro and nano-sized zirconia oxide particles were tested in which both groups showed improved radiopacity property as recommended by ISO/ADA standards of 3 mm Al [42].

#### Tio2 nanoparticles

Titanium dioxide NPs are highly stable particles with suitable photocatalytic properties. It causes Oxidative stress due to the generation of reactive oxygen species. There is superior membrane fluidity and cell membrane disruption due to its property of lipid peroxidation. It is also used as an effective antifungal for fluconazole-resistant strains [43–45].

#### MgO and CaO nanoparticles

These NPs are found to be capable against both Gram-positive and Gram-negative microorganisms. Their antibacterial activity is for the reason that they disrupt the cell membrane leading to leakage of intracellular contents and eventually cell death [46, 47].

A study conducted by Kishen et al. comparing 5.25% NaOCl, MgO Nanoparticles at a concentration of 5 mg/L and Chitosan Nanoparticles to compare their long lasting efficacy in the eradication of *Enterococcus faecalis*. It was concluded that both Magnesium oxide and Chitosan nanoparticles showed comparable or superior results to the gold standard 5.25% sodium hypochlorite irrigant [24].

#### CuO nanoparticles

These nanoparticles are effective against Gram positive and gram negative bacteria as they cross the bacterial cell membrane and damage the vital enzymes of the bacteria. They also possess certain antifungal properties. However, their application in the field of endodontics is limited and further studies are required to evaluate its efficacy [48, 49].

The commonly used nanoparticles in endodontics are summarized in Table 1.

**Table 1:** Summarization of properties of commonly used nanoparticles in Endodontics

Nanoparticle	Mechanical property	Physical property	Chemical property	Biological property
Graphene	1. highly stable 2. transparent 3. flexible material 4. increased ductility and malleability	1. High surface area is due to its peculiar structure. 2. Excellent electronic properties 3. Excellent optical properties.	1. Presence of a 2D structure comprising of single, thick carbon sheets arranged in a honeycomb pattern.	1. Good antimicrobial properties especially against S.mutans 2. Enhanced tissue dissolving properties. 3. Low toxicity level
Carbon nanotubes	1. Higher tensile strength as they have a hexagonal arrangement 2. Malleability is comparable to that of rubber 3. High ductility (8–12%) 4. Superior mechanical strength.	1. Large Surface area 2. Extremely light weight 3. Highly heat stable 4. Low density.	1. Good conduction efficiency 2. Superior bonding between these atoms making these NPs quite stable 3. Carbon atoms are arranged in the form of hexagonal rings	1. Enhanced antimicrobial properties 2. Ability to penetrate the bacterial cell membrane. 3. Induces inflammatory and fibrotic reactions under extreme conditions. 40 Potentially toxic in nature.
Silver nanoparticles	1. Good conductors of electricity 2. Possessing good malleability and ductility.	1. Due to its small size and high surface it confers excellent electrical, optical, thermal properties	1. Enhanced surface chemistry thereby making it an effective antibacterial agent	1. Effctive antimicrobial agent especially against E.faecalis. 2. Increased permeability in the bacterial cell membrane. 3. Highly biocompatible 4. Low toxicity levels.
Chitosan	1. Inactive and non-soluble in water alkali and organic solvents 2. pH more than 6	1. Soluble in various other mediums 2. Highly viscous, with a polyelectrolyte property.	1. It is a linear polyamine. 2. The presence of highly reactive hydroxyl and amino groups results in chelation of various transitional metal ions [38].	1. Excellent antibacterial, antifungal and antiviral properties. 2. Causes disruption of the bacterial cell membrane due to its electrostatic interaction.

## Future of nanoparticles in endodontics

Nanotechnology has presented is impression on almost every field of science and development. Naturally, medicine and dentistry too have been inspired by this technology having an enormous potential. This being said, there is no doubt that the future of endodontics is heading down the nano-direction as most of the challenges faced (microorganisms, dentin) are all nano- sized. The era of nano-endodontics is paving it’s way to be the bright future in dentistry.

## Conclusion

The influence of Nanoparticles in the field of dentistry and especially endodontics for the treatment of various oral diseases is rapidly progressing with every passing year. Nanomaterials (NMs) have recently gained importance in technological advancements due to their superior physical, mechanical, chemical and biological properties. These properties have resulted in better performance as compared to that of their conventional counterparts.

Nanomaterials have shown great promise to reduce biofilm formation, enhance remineralization of the tooth structure by inhibiting its demineralization process, and to counteract the caries-related and endodontic microorganisms. These results have been inspiring enough to open the doors for further clinical studies that will allow the therapeutic value of nanotechnology-based materials to be authenticated.

## Data Availability

Data sharing is not applicable to this article as no datasets were generated or analysed during the current study.

## Abbreviations

EPS: Extracellular polymeric substances
nm: Nanometer
NPs: Nanoparticles
ROS: Reactive oxygen species
ATP: Adenosine tri phospahte
CHX: Chlorhexidine
EDTA: Ethylenediaminetetraacetic acid
NaOCl: Sodium hypochlorite
GP: Gutta percha
NDGP: Nano-diamond GP
AgNP’s: Silver Nanoparticles
Hap: Hydroxyapatite
ZrO2: Zirconia Oxide
FeOx: Iron Compound
MTA: Mineral trioxide aggregate
ISO: International Organization for Standardization
ADA: American Dental Association
SiO_2_, Na_2_O, P_2_O_5,_ CuO: Silicon di oxide, Sodium oxide, Phosphorus Pentoxide, Copper oxide
Tio2 nanoparticles: Titanium di oxide
MgO and CaO: Magnesium oxide and Calcium oxide
NMs: Nanomaterials
